# Sequence–Activity Relationships for the Snf7 Insecticidal dsRNA in Chrysomelidae

**DOI:** 10.3389/fpls.2020.01303

**Published:** 2020-08-25

**Authors:** Pamela Bachman, Jennifer Fridley, Geoffrey Mueller, William Moar, Steven L. Levine

**Affiliations:** ^1^Science Organization, The Climate Corporation, Creve Coeur, MO, United States; ^2^Regulatory Science, Bayer Crop Science, Chesterfield, MO, United States

**Keywords:** RNAi, dsRNA, insecticidal, ortholog, Diabrotica, Leptinotarsa, non-target organism

## Abstract

The responsiveness of insects to oral delivery of insecticidal dsRNA has been shown to be dependent on dsRNA length and sequence match. Previous work with the western corn rootworm (WCR, *Diabrotica virgifera virgifera*; Coleoptera: Chrysomelidae) demonstrated that at least one ≥21 nt match must be present in the DvSnf7 dsRNA of approximately ≥60 base-pairs (bp) for activity. Further data is needed on the activity of <21 nt matches along with characterization of relationship between activity and the number of ≥21 nt matches. To characterize the sequence–activity relationship for insecticidal dsRNA further, the activity of orthologous Snf7 dsRNAs with 19, 20, and 21 nt contiguous matches against WCR was compared. Neither 19 nor 20 nt sequence matches were active, supporting that a ≥21 nt sequence match is required for activity. The relationship between the number of 21 nt matches with activity of Snf7 dsRNA orthologs from several Chrysomelid species was characterized using WCR and Colorado potato beetle (CPB, *Leptinotarsa decemlineata;* Coleoptera Chrysomelidae). For WCR, there was a strong relationship between an increasing number of 21 nt matches and increased activity (*i.e.*, lower LC_50_ values). A similar relationship was observed for CPB with an exception for a single ortholog, which may be related to the exceptionally high rate of polymorphisms in CPB. Overall, these results demonstrate a general relationship between the number of 21 nt matches and activity, and this relationship could be used to inform a testing and assessment plan for an ecological risk assessment for an insecticidal dsRNA.

## Introduction

The registration of genetically engineered (GE) plants that express double-stranded RNA (dsRNA) to control insect pests through RNA interference (RNAi) has brought a new mode of action (MOA) for pest control to the market. The efficacy and its potential for an improved environmental safety profile of such products have gained interest from academic, government, and private sector researchers. Several factors including exposure concentration, potency, sequence and length, time-to-effect, persistence of gene silencing, and the insect life-stage, have been identified as influencers on efficacy against the target (Huvenne and Smagghe, 2010; Bolognesi et al., 2012; Bachman et al., 2013).

In general, long dsRNAs that incorporate a high degree of sequence match to mRNAs in the target insect have greater potential for potency as a result of the number of siRNAs that can be produced from the sequence of each long dsRNA (Baum et al., 2007; Miller et al., 2012; Haller et al., 2019). Bolognesi et al. (2012) and Miller et al. (2012) demonstrated that a dsRNA must be of sufficient length (*e.g.* ≥60 bp) to result in activity against western corn rootworm (WCR; *Diabrotica virgifera virgifera*) and *Tribolium castaneum*, respectively. Additionally, Bolognesi et al. (2012) demonstrated that a single 21 nucleotide (nt) contiguous sequence match in ≥60 bp was sufficient for southern corn rootworm (SCR, *Diabrotica undecimpunctata howardi*) activity. In comparison, Bachman et al. (2013) demonstrated that a single 19 nt contiguous match in a 240 bp Snf7 orthologous dsRNA did not have activity against WCR in diet bioassays. However, a gap exists with empirical data to address the potential activity of a single 20 nt contiguous match, and there was a need to further characterize the relationship between the number of 21 nt contiguous sequence matches and activity. Further, as demonstrated in Miller et al. (2012), the potency of a dsRNA is positively related to the number of potential 21 nt matches contained in the sequence, and therefore the number of 21 nt matches should be considered within the ecological risk assessment (ERA) and relevant environmental exposure necessary for activity under realistic exposure scenarios for non-target organism (NTOs) in the agroecosystem.

Here we provide additional bioassay data to address these gaps and further characterize the response of insects to environmental (exogenous) dsRNA. Efforts were focused on the beetles from the family Chrysomelidae (chrysomelids) because they have become well established models for these types of bioassays due to their responsiveness to oral delivery of dsRNA and are the target of the first registered insecticidal dsRNA product.

## Material and Methods

A series of bioassays were performed using two chrysomelid beetles, WCR and the Colorado potato beetle (CPB; *Leptinotarsa decemlineata*) with established methods for laboratory testing. Bioassays were conducted using diet-incorporation, and the test arthropods were fed dsRNA in diets *ad libitum*. Bioassays followed published methods described in Bachman et al. (2013) using 240 bp dsRNAs, DvSnf7_240 dsRNA targeting WCR and LdSnf7 dsRNA (240 bp) targeting CPB. Additional 240 bp Snf7 orthologous dsRNA sequences for testing were chosen from an internal library of insect sequences and selected for the number of 21 nt matches as compared back to the DvSnf7_240 and LdSnf7 dsRNAs as described in Bachman et al. (2013; **Supplementary Material S1**). dsRNAs were prepared as described in Bolognesi et al. (2012) and Bachman et al. (2013) with the MEGAscript kit (Ambion) following the manufacturer’s protocol. Purified dsRNAs were quantified by spectroscopy and examined by agarose gel electrophoresis to ensure their integrity.

### Examination of Minimum Sequence Necessary for Biological Activity

Single dietary concentrations of three dsRNA treatments were tested concurrently at limit concentrations in 12-day diet bioassays to determine if a dsRNA containing single 19 nt, 20 nt, or 21 nt contiguous matches to the DvSnf7_240 dsRNA have a biological effect on larval WCR. The single 19 nt match was identified in the Snf7 240 bp dsRNA ortholog from *Chrysolina quadrigemina* (CqSnf7) and was previously fed to WCR at 5,000 ng/ml diet with no significant impact on survival (Bachman et al., 2013) and served as the negative (no activity) control. A single 20 nt match was identified in the Snf7 240 bp ortholog from *Drosophila pseudoobscura* (DpSnf7) and has not been previously evaluated against WCR or SCR. The DvSnf7_21.7 dsRNA construct consists of a single 21 nt match to the DvSnf7 dsRNA embedded in a neutral dsRNA carrier (240 bp total length) and served as the positive control. Previously, single 21 nt match dsRNA constructs, such as DvSnf7_21.7 dsRNA, were shown to induce significant mortality in SCR but were tested at concentrations at least an order of magnitude greater than the full length DvSnf7_240 dsRNA (Bolognesi et al., 2012).

For each bioassay, three replicates of a single treatment targeting 1,000 ng/ml were prepared for the CqSnf7 and DpSnf7 dsRNA test substances. The DvSnf7_21.7 dsRNA treatment was conducted at a single concentration level prepared at 374 ng/ml. Based upon the known response of SCR to DvSnf7_21.7 dsRNA and the known differences between WCR and SCR response to the DvSnf7_240 dsRNA in our laboratory, the concentration of 374 ng/ml assured a positive response in WCR (Bolognesi et al., 2012). Additionally, three water (diet only) replicates were prepared as an assay control for each bioassay. For the assessment of the minimum sequence necessary for activity, differences for insect mortality between treatments were evaluated with a generalized linear model and a binomial distribution under PROC GLIMMIX, and differences in insect mass between treatments were evaluated with a linear model under PROC MIXED (SAS, 2012). Pairwise comparisons were made between each treatment and the assay control for survival and mass using t-tests. All significance tests were determined at the 0.05 level.

### Examination of Number of 21 nt Matches Influence on Biological Activity

To examine the influence of the number of 21 nt matches on activity, orthologous Snf7 dsRNAs from a selection of chrysomelid beetles were fed to WCR and CPB in 12-day diet bioassays to characterize the concentration–response relationship and to estimate LC_50_ values (**Table 1**). dsRNAs lacking 21 nt matches were tested at a single limit dose of 5,000 ng/ml. Along with the DvSnf7_240 dsRNA, orthologous dsRNAs from four species closely related to WCR were utilized in these assays; *Acalymma vittatum (AvSnf7), Cerotoma trifurcata (CtSnf7), Galerucella calmariensis (GcSnf7)*, and *Chrysolina quadrigemina (CqSnf7)*. These orthologs were previously described in Bachman et al. (2013) and evaluated for sequence alignment against DvSnf7_240 dsRNA and biological activity at a single high concentration. *A. vittatum, C. trifurcata*, and *G. calmariensis* were demonstrated to contain 21 nt matches to DvSnf7 and to be active against WCR, whereas *C. quadrigemina* was shown to lack any 21 nt matches and activity against WCR (Bachman et al., 2013). Concentration–response bioassays and synthesis of the dsRNAs used in these bioassays followed the method described in Bachman et al. (2013). For CPB, orthologous dsRNAs from *Microtheca ochroloma* (MoSnf7) and *Aphthona lacertosa* (AlSnf7) were tested in addition to the LdSnf7, CqSnf7 and GcSnf7 dsRNAs. LC_50_ values were estimated by logistic regression with responses that were corrected for control mortality (Van Ewijk and Hoekstra, 1993).

## Results

### Examination of Minimum Sequence Necessary for Biological Activity

Mean WCR survival was 80, 83, 82, and 29% for the water only control, CqSnf7, DpSnf7 and DvSnf7_21.7 dsRNA single dietary concentration treatments, respectively. Mean mass for WCR was 0.32, 0.31, 0.32, and 0.22 mg, for the water only control, CqSnf7, DpSnf7 and DvSnf7_21.7 dsRNA positive control treatment, respectively. There was no significant difference in survival and mean mass between either the CqSnf7 and DpSnf7 treatments *versus* the control (p > 0.05), whereas there was a highly significant difference between survival and mean mass in the DvSnf7_21.7 dsRNA *versus* the water-only control (p < 0.001). Results from these assays demonstrate that continuous dietary exposure to dsRNAs containing only 19 or 20 nt contiguous matches have no effect on survival or growth of WCR. Decreased survival and inhibited growth of WCR larvae fed the DvSnf7_21.7 dsRNA was consistent with previous reports (Bolognesi et al., 2012; Bachman et al., 2013).

### Examination of Number of 21 nt Matches Influence on Biological Activity

**Figure 1:**
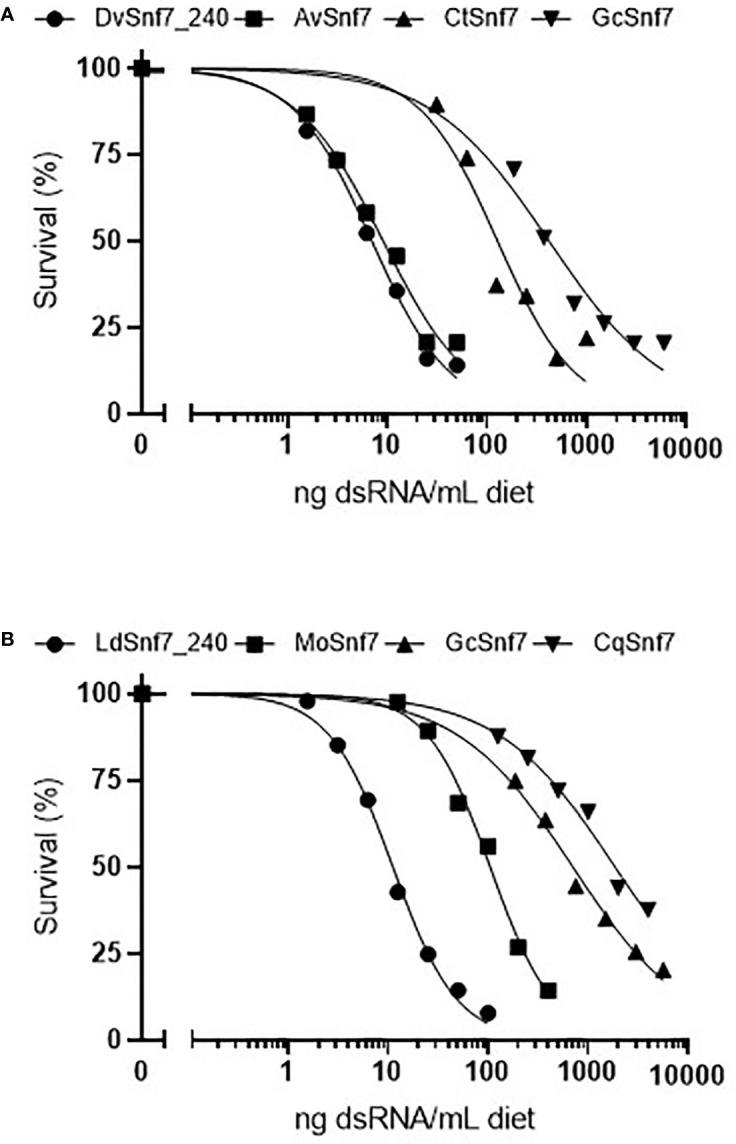
Concentration–response curves with orthologous dsRNAs demonstrate that the activity of dsRNA with **(A)** the western corn rootworm (WCR, *Diabrotica virgifera virgifera*) (WCR) and **(B)** the Colorado potato beetle (CPB, *Leptinotarsa decimlineata*) is related to the degree of shared sequence including the number of potential 21 nt matches. Survival in the water only controls for the DvSnf7, AvSnf7, CtSnf7, LdSnf7, MoSnf7, GcSnf7, and CqSnf7 treatments were 92, 92, 83, 81, 92, 93, 88, and 99%, respectively. Data is reported and analyzed as corrected for control mortality. Concentration–response curves and LC_50_ estimates were performed in Prism GraphPad v8.2.0.

To characterize the relationship between the number of 21 nt matches in a dsRNA sequence and activity, concentration–response bioassays were performed with dsRNAs that contained possible 21 nt matches against WCR and CPB in 12-day diet bioassays (**Figures 1A, B**). Where dsRNAs lacked 21 nt matches, tests were conducted at a single limit dose, and significant mortality (p > 0.05) was not observed when these sequences were fed to WCR and CPB (**Table 1**). Results from WCR, demonstrate a strong positive relationship between an increasing number of 21 nt matches and mortality (**Table 1**; **Figure 1A**). However, for CPB, there was only a general relationship between the number of 21 nt matches and mortality (**Table 1**; **Figure 1B**); the lowest LC_50_ value was observed for the LdSnf7 (11.2 ng/ml), which had a 240 bp (*i.e.*, 100%) contiguous sequence match with the CPB *snf7* gene. However, two different orthologs each with 12 possible 21 nt matches had a large difference in activity (105 ng/ml *vs.* 1860 ng/ml), and a fourth ortholog with 3 possible 21 nt matches had an LC_50_ value that was between the values for the orthologs with 12 possible 21 nt matches (704 ng/ml). Overall, these results are largely consistent with the findings of Miller et al. (2012) with the beetle *T. castaneum*, where greater activity was observed with longer dsRNAs with a contiguous sequence match (*e.g.* 520 bp) injected into larvae *versus* shorter dsRNAs with a contiguous sequence match (*e.g.* 69 bp). Similar results demonstrating the relationship between the number of 21 bp matches and activity was reported with the WCR and dietary exposure (Bolognesi et al., 2012).

**Table 1:** Relationship between the number of 21 nt matches and activity based on concentration–response bioassays performed with dsRNAs that contained zero to 221 possible 21 nt matches against WCR and CPB in 12-day diet bioassays.

dsRNA ortholog Source Species, Subfamily	WCR LC_50_ and 95% confidence interval (ng/ml)	% Shared identity with DvSnf7_240	# 21 nt matches	Longest contiguous nt sequence(s)
*Tested against WCR*				
DvSnf7 *Diabrotica virgifera virgifera*; (WCR) **Galerucinae**	7.0 (6.0–8.1)	100	221	240
AvSnf7 *Acalymma vittatum* **Galerucinae**	9.1 (7.3–11.4)	95.0	69	38, 38, 47, 26
CtSnf7 *Cerotoma trifurcata* **Galerucinae**	128 (80–210)	90.8	18	32, 26
GcSnf7 *Galerucella calmariensis* **Galerucinae**	421 (239–632)	90.8	3	23
CqSnf7 *Chrysolina quadrigemina* **Chrysomelinae**	No Activity	82.1	0	19
*Tested against CPB*				
LdSnf7 *Leptinotarsa decimlineata* (CPB) **Chrysomelinae**	11.2 (9.9–12.6)	100	221	240
MoSnf7 *Microtheca ochroloma* **Chrysomelinae**	105 (90–123)	84.2	12	32
CqSnf7 *Chrysolina quadrigemina* **Chrysomelinae** GcSnf7 *Galerucella calmariensis* **Galerucinae**	1,860 (1,496–2,406) 704 (584–841)	87.5 80.8	12 3	23, 29 23
AlSnf7 *Aphthona lacertosa* **Galerucinae**	No Activity	77.1	0	14

Significant mortality (p > 0.05) was not observed when dsRNA sequences lacking 21 nt matches were fed to WCR and CPB. dsRNAs lacking 21 nt matches were tested at a single limit dose of 5,000 ng/ml. For WCR, survival in the CqSnf7 treatment was 88%, and survival in the water-only control was 94%. For CPB survival in the AlSnf7 treatment was 93%, and survival in the water-only control was 91%.

## Discussion

In cases where responsiveness to environmental dsRNA and the presence of 21 nt matches are known for an NTO, understanding the relationship between the degree of sequence match and activity can inform an ERA and testing plan that evaluates the potential for adverse effects in NTOs. Haller et al. (2019) fed dsRNAs with multiple 21 nt matches to NTO ladybeetles, *Adalia bipunctata*, and *Coccinella septempunctata*. Activity against these two species was predicted, based upon previous confirmation of responsiveness in ladybeetles, and confirmed in dietary bioassays. Additionally, Haller et al. (2019) reported on the difference in response of these two species in regard to the number of 21 nt matches present, with *C. septempunctata* (34 matches) showing more responsiveness than *A. bipunctata* (six matches). These results are consistent with our findings here, where the potency of the dsRNA with 21 nt matches was related to the number of 21 nt matches with one exception. The reason why comparatively low activity was observed for one of the two CPB orthologs with 12 possible 21 nt matches is unclear. However, one explanation for the differences in activity between these two orthologs may be related to the exceptionally high rate of polymorphisms in CPB relative to vertebrates and other beetles (Schoville et al., 2018). Therefore, CPB was not an ideal species to characterize the relationship between the number of 21 nt matches and activity. Combined with an exposure assessment (*e.g.* environmental concentration of the dsRNA), this general relationship can be used predictively in an ERA for responsive species to aid in an understanding of potential adverse effects to NTOs be assessed to meet the protection goals (*e.g.* population level effects for a valued NTO).

Further, we have demonstrated that for WCR, dsRNAs with only 19 nt or 20 nt matches are not active and that a minimum of a 21 nt sequence match is necessary for activity of a long (*e.g.* ≥60 bp) dsRNA. This information can also be used to inform an ERA, especially in regard to the selection of NTOs for evaluation. Bachman et al. (2013) suggested that when bioinformatics data for NTOs is available and indicates that the minimum sequence requirements for dsRNA activity (<21 nt matches) are not met, toxicity testing may not be necessary as the likelihood of adverse effects is low. The activity of DvSnf7 dsRNA against WCR was demonstrated to follow phylogeny within Chrysomelida with ≥21 nt matches not found outside the family Chrysomelidae (Bachman et al., 2013). Tan et al. (2016) compared sequence analysis and toxicity testing with the NTO, honey bee (*Apis mellifera:* Hymenoptera: Apidae), and the DvSnf7 dsRNA. No ≥21 nt matches exist between the DvSnf7 dsRNA and the honey bee ortholog leading to a prediction of no toxicity, and laboratory bioassays with both adult and larval honey bee at high concentrations confirmed the predicted lack of activity. Further NTO screening with taxa (outside of Chrysomelidae) representing different ecological receptors (*e.g.* pollinators, detritivores, natural enemies, vertebrates) and a bioinformatics screening of NTOs associated with maize agroecosystems confirmed the lack of activity and lack of ≥21 nt matches beyond Chrysomelidae (Bachman et al., 2016). While these data were used to inform the ERA for the DvSnf7 dsRNA-expressing maize product and supported the conclusion of negligible risk to the environment (Bachman et al., 2016; U.S. EPA, 2017), some species could be eliminated from testing as no mechanism exists for an RNAi effect to occur.

While identifying the presence or absence of 21 nt matches can be used to predict activity in species known to be responsive to environmental dsRNA, it is not a reliable standalone tool for predicting activity in species that have unknown responsiveness or are recalcitrant to environmental dsRNA. Multiple studies with arthropods ranging from springtails (Pan et al., 2016) to lepidoptera (Pan et al., 2017) have demonstrated no adverse effects of environmental dsRNA even in the presence of a high degree of sequence match. The lack of responsiveness is likely linked to biological barriers such as nucleases in saliva or hemolymph that degrade the dsRNA or inefficient uptake mechanisms from these arthropod’s midguts (Baum and Roberts, 2014; Ivashuta, 2015).

## Data Availability Statement

The datasets generated for this study are available on request to the corresponding author.

## Author Contributions

PB and SL conceived the studies and designed the experiments. JF and GM performed the experiments and SL analyzed the results. PB, WM, and SL conceptualized and wrote the manuscript. All authors contributed to the article and approved the submitted version.

## Funding

The research reported was funded by Bayer Crop Science and the researchers involved in this work were employees of Bayer Crop Science and its predecessors in business.

## Conflict of Interest

All authors are Bayer employees, and Bayer accepted no outside funding for this research represented in this manuscript. The funder (Bayer and its predecessors in business) provided support in the form of salaries for all authors, and also played a role in the design, data analysis and the decision to publish.
