# Supplementary material for: Sequence–Activity Relationships for the Snf7 Insecticidal dsRNA in Chrysomelidae
Source: Front Plant Sci. 2020 Aug 25;11:1303. doi: 10.3389/fpls.2020.01303 (PMC7477086; doi:10.3389/fpls.2020.01303)
Supplement: Supplementary file 1 [file Table_1.docx]

**Supplementary Material for** **Sequence-activity relationships for the Snf7 insecticidal dsRNA in Chrysomelidae**

**S1**

Snf7 orthologous dsRNA sequences (240 bp) used for testing against WCR and CPB. dsRNAs were prepared as described in Bolognesi et al. (2012) and Bachman et al. (2013) with the MEGAscript kit (Ambion) following manufacturer’s protocol.  Purified dsRNAs were quantified by spectroscopy and examined by agarose gel electrophoresis to ensure their integrity.

Western Corn Rootworm Series

Western Corn Rootworm, *Diabrotica virgifera virgifera*; DvSnf7 dsRNA

GCAAAGAAAAATGCGTCGAAAAATAAAAGAGTTGCACTCCAAGCCCTCAAAAAGAAGAAACGATTGGAAAAGACCCAACTACAAATAGATGGAACCCTTACAACTATTGAAATGCAGAGGGAAGCCCTCGAAGGAGCTAGCACAAATACTGCTGTATTAGATTCTATGAAAAATGCTGCAGATGCCCTTAAGAAAGCTCATAAGAATTTGAATGTAGATGATGTTCACGATATCATGGAT

Other Chrysomelid dsRNAs tested against WCR. Matches to the DvSnf7 dsRNA are in bold text. Single nucleotide polymorphisms (SNPs) are unbolded and underlined. The number of possible 21 nucleotide (nt) matches SNPs are also provided.

*Acalymma vittatum*; AvSnf7 dsRNA; 69 possible 21 nt matches, 12 SNPs

**GCAAAGAAAAATGCGTCGAAAAATAAAAGAGTTGCACT**A**CAAGC**A**CTCAAAAAGAAGAAACGATTGGAAAAGACCCAACTACA**G**ATAGATGGAAC**T**CTTACAAC**G**ATTGAAATGCAGAGGGAAGCCCTCGAAGGAGCTAGCACAAATACTGC**A**GTATTAGATTCTATGAA**G**AATGCTGCAGATGCCCTTAAGAAAGC**A**CATAAGAA**C**TTGAATGT**G**GATGATGTTCA**T**GA**C**ATCATGGAT**

*Cerotoma trifurcata*; CtSnf7 dsRNA; 18 possible 21 nt matches, 22 SNPs

**GCAAAGAAAAATGC**A**TCGAAAAATAAAAGAGT**G**GCACT**T**CAAGC**T**CTCAAAAAGAAGAA**G**CG**G**TTGGAAAAGACCCAACTACAAATAGATGGAAC**A**CTTACAAC**A**ATTGAAATGCAGAG**A**GA**G**GC**G**CT**G**GAAGGAGCTAG**T**ACAAATACTGCTGTATT**G**GATTCTATGAAAAATGCTGCAGATGC**A**CTTAAGAAAGC**C**CATAAGAA**CC**T**A**AATGT**G**GATGATGTTCA**T**GATAT**T**ATGGAT**

*Galerucella calmariensis*; GcSnf7 dsRNA; 3 possible 21 nt matches, 22 SNPs

**GCAAAGAAAAATGC**A**TC**A**AAAAATAAAAGAGTTGcACT**T**CAAGCCCT**T**AAAAAGAAGAAA**A**GATTGGAAAAGA**A**CCAA**T**T**G**CAAATAGATGGAACCcTTACAAC**A**ATTGAAATGCAG**C**G**T**GAAGC**T**CTCGAAGGAGC**c**AGCACAAATAC**A**GCTGTATTAGATTC**A**ATGAAAAATGCT****gCAGATGC**A**CT**C**AAGAAAGCTCATAA**A**AA**C**TTG**G**ATGT**G**GATGATGTTCA**T**GATATCATGGAT**

*Chrysolina quadrigemina*; CqSnf7 dsRNA; No possible 21 nt matches, 43 SNPs

**GC**T**AAGAAAAATGC**A**TCGAAAAA**C**AAAAGAGT**G**GC**T**CT**T**CAAGC**T**CT**T**AA**G**AA**A**AAGAAACGATTGGAAAAGA**AT**CA**G**CT**G**CAAATAGATGGGAC**T**CT**A**ACAAC**C**ATTGAA**C**T**T**CA**AC**G**A**GAAGC**T**CTCGA**G**GGAGC**C**AGCAC**G**AA**C**AC**GA**C**G**GTATTAGA**A**TCTATGAAAAATGC**A**GC**T**GA**A**GC**T**CT**C**AA**A**AAAGCTCATAA**A**AATTTG**G**ATGT**T**GAT**A**ATGTTCACGA**C**AT**T**ATGGAT**

Colorado Potato Beetle Series

Colorado Potato Beetle, *Leptinotarsa decemlineata*; LdSnf7 dsRNA

GCAAAGAAAAATGCATCAAAAAACAAAAGAGTGGCCCTGCAAGCCCTCAAGAAAAAGAAGCGATTAGAAAAAAATCAGTTGCAAATAGATGGCACTTTAACTACTATTGAACTTCAACGAGAAGCTCTGGAGGGAGCTAGTACGAACACCACAGTATTGGAATCTATGAAAAATGCAGCTGAAGCTCTTAAGAAAGCCCATAAAAACTTGGACGTGGACAATGTGCATGACATCATGGAT

Other Chrysomelid dsRNAs tested against CPB. Matches to the LdSnf7 dsRNA are in bold text. Single nucleotide polymorphisms (SNPs) are unbolded and underlined. The number of possible 21 nucleotide (nt) matches SNPs are also provided.

*Microtheca ochroloma*; MoSnf7 dsRNA; 12 possible 21 nt matches, 36 SNPs

**GC**C**AAGAAAAATGC**T**TCAAAAAA**T**AA**G**AGAGTGGC**T**CT**A**CA**G**GC**A**TT**G**AAGAAAAAGAA**A**CGATT**G**GAAAA**G**AATCAGTTGCAAAT**C**GATGG**A**AC**A**TTAAC**A**AC**C**ATTGAACTTCA**G**CGAGAAGC**A**CT**T**GAGGGAGCTAG**C**AC**T**AA**T**AC**T**ACAGT**T**TT**A**GAATCTATGAAAAATGCAGCTGAAGCTCTTAA**A**AAAGC**T**CATAAAAA**T**CT**T**GA**T**GT**T**GA**T**AATGT**A**CA**C**GACAT**A**ATGGAT**

*Chrysolina quadrigemina*; CqSnf7 dsRNA; 12 possible 21 nt matches, 30 SNPs

**GC**T**AAGAAAAATGCATC**G**AAAAACAAAAGAGTGGC**T**CT**T**CAAGC**T**CT**T**AAGAAAAAGAA**A**CGATT**G**GAAAA**G**AATCAG**C**TGCAAATAGATGG**G**ACT**C**TAAC**A**AC**C**ATTGAACTTCAACGAGAAGCTCT**C**GAGGGAGC**C**AG**C**ACGAACAC**G**AC**G**GTATT**A**GAATCTATGAAAAATGCAGCTGAAGCTCT**C**AA**A**AAAGC**T**CATAAAAA**T**TTGGA**T**GT**T**GA**T**AATGT**T**CA**C**GACAT**T**ATGGAT**

*Galerucella calmariensis*; GcSnf7 dsRNA, 3 possible 21 nt matches, 46 SNPs

**GCAAAGAAAAATGCATCAAAAAA**T**AAAAGAGT**T**Gc**A**CT**T**CAAGCCCT**T**AA**A**AA**G**AAGAA**AA**GATT**G**GAAAA**G**AA**C**CA**A**TTGCAAATAGATGG**A**AC**Cc**T**T**AC**A**AC**A**ATTGAA**A**T**G**CA**G**CG**T**GAAGCTCT**C**GA**A**GGAGC**c**AG**C**AC**A**AA**T**AC**AG**C**T**GTATT**A**GA**T**TC**A**ATGAAAAATGC**T**gC**A**GA**T**GC**A**CT**C**AAGAAAGC**T**CATAAAAACTTGGA**T**GTGGA**TG**ATGT**T**CATGA**T**ATCATGGAT**

*Aphthona lacertosa*; AlSnf7 dsRNA; No possible 21 nt matches, 55 SNPs

**GC**T**AA**A**AA**G**AATGCATC**T**AA**G**AA**T**AAAAG**G**GTGGCCCT**A**CA**G**GC**T**CT**T**AA**A**AA**G**AAGAAG**A**GATT**G**GAAAA**G**A**C**TCAG**C**T**A**CAAAT**C**GATGG**A**AC**AC**T**T**AC**A**AC**C**ATTGAA**A**T**G**CA**G**CG**G**GA**G**GC**C**CT**C**GA**A**GGAGCTAGTAC**C**AACAC**TG**CAGT**T**TT**A**GA**T**TCTATGAAAAA**C**GC**T**GC**A**GA**T**GC**A**CT**G**AA**A**AAAGC**A**CA**C**AAAAACTTGGACGT**C**GACAA**C**GT**T**CA**C**GACAT**T**ATGGA**C
